# Early Mobilization Dose Reporting in Randomized Clinical Trials With Patients Who Were Mechanically Ventilated: A Scoping Review

**DOI:** 10.1093/ptj/pzae048

**Published:** 2024-03-22

**Authors:** Felipe González-Seguel, Renato Letelier-Bernal

**Affiliations:** School of Physical Therapy, Faculty of Medicine, Clínica Alemana Universidad del Desarrollo, Santiago, Chile; Programa de Magíster en Fisiología Clínica del Ejercicio, Facultad de Medicina y Ciencias de la Salud, Universidad Mayor, Santiago, Chile; Programa de Magíster en Fisiología Clínica del Ejercicio, Facultad de Medicina y Ciencias de la Salud, Universidad Mayor, Santiago, Chile

**Keywords:** Clinical Trials, Early Mobilization, Exercise, Intensive Care Unit, Rehabilitation

## Abstract

**Objective:**

The aim of this scoping review was to investigate the mobilization dose reporting in the randomized clinical trials (RCTs) of patients receiving mechanical ventilation in the intensive care unit.

**Methods:**

In this scoping review, RCTs published from inception to December 2022 were searched in relevant electronic databases. Trials that involved adults receiving mechanical ventilation (>48 hours) and any early mobilization modality were analyzed. Two independent authors screened, selected, and extracted data. The mobilization doses of the intervention groups (IGs) and the comparator groups (CGs) were assessed as the proportion of reported items/total applicable from the main items of the Consensus on Exercise Reporting Template (CERT).

**Results:**

Twenty-three RCTs comprising 2707 patients (1358 from IG and 1349 from CG) were included, involving studies on neuromuscular electrical stimulation (*n* = 7), progressive mobility (*n* = 6), leg cycling (*n* = 3), tilt table (*n* = 1), and multicomponent (*n* = 6) mobilization. The pooled reporting of CERT items was 68% (86% for IG and 50% for CG). The most reported CERT items were type of exercise (100%) and weekly frequency (100%) for IG, whereas the least reported were intensity (4%) and individualization (22%) for CG. Regardless of the group, individualization, progression, and intensity of mobilization were the least reported items. Eight IGs (35%) reported all CERT items, whereas no CGs reported all of them.

**Conclusions:**

Deficits in mobilization dose reporting of intensive care unit RCTs were identified, especially for exercise intensity in adults receiving mechanical ventilation. One-third of IG reported all exercise dosing items, whereas no CG reported all of them. Future studies should investigate the details of optimal dosage reporting, particularly for CG.

**Impact:**

The lack of dose reporting may partially explain the inconsistency in the meta-analysis results of early mobilization trials, thus limiting the interpretation for clinical practice in the intensive care unit.

## Introduction

Approximately 30 to 90% of intensive care unit (ICU) patients worldwide receive mechanical ventilation.^1^ As patients receiving mechanical ventilation become inactive for long periods,^2^^,^^3^ their participation in out-of-bed activities in the ICU is limited,^4–8^ therefore there is a risk of physical, mental, and cognitive ICU-related consequences.^9^^,^^10^ These consequences are part of the post–intensive care syndrome: ICU-acquired weakness, delirium, anxiety, and depression.^11^^,^^12^ Recommended strategies to minimize long-term consequences of the ICU have been established in relevant guidelines and bundles on pain, agitation/sedation, delirium, ventilatory weaning, immobility/mobilization, family engagement, and sleep disruption.^13–15^ Specifically, early mobilization has been a significant rising trend,^16^ established as part of the ICU standard of care,^13^ where mobilization protocols usually include progressive mobility, passive or active exercises, cycling, neuromuscular electrical stimulation (NMES), and functional electrical stimulation. However, despite the global implementation of early mobilization,^17^ patients receiving mechanical ventilation have poor capacity to perform active mobilization (16% of the time in the ICU) and infrequently progressing to walking (4% of the time in the ICU).^5^^,^^8^

Early mobilization decreases ICU and hospital length of stay,^18^ thereby improving physical functioning at 6 months^19^ and cognitive functioning at 12 months.^20^ However, significant changes on muscle strength, duration of mechanical ventilation, adverse events, survival, and health-related quality of life by early mobilization have not yet been confirmed in comparison with those by usual care.^18^^,^^19^^,^^21^ A large number of randomized clinical trials (RCTs) have pursued to demonstrate the effectiveness of early mobilization in patients in the general ICU^18^^,^^19^ and in patients receiving mechanical ventilation.^21^^,^^22^ Considering that the RCT is the gold standard study for the evaluation of health interventions and the second level of evidence for clinical decision making,^23^ the contradictory findings of RCTs on ICU mobilization have been described as a frail evidence base,^24^ deserving discussion in last years.^17^

The coexistence of “positive” and “non-positive” effects of early mobilization in the ICU setting have strengthened evidence gaps on dosing, timing, and safety of the intervention.^24^ Specifically, the intervention delivered in the intervention groups (IG) of some landmark early mobilization RCTs has not been delivered as planned, guiding to the intervention fidelity concept.^25^^,^^26^ Some comparator groups (CG) have reached the same or even higher mobilization level compared with IG.^26^^,^^27^ Therefore, the lack of tracking and reporting of the mobilization dosing of the RCTs could limit the fidelity of the planned interventions. Hence, the aim of this scoping review was to investigate the ICU mobilization dose reporting in the RCTs for patients receiving mechanical ventilation.

## Methods

### Study Design

This scoping review was performed according to the Joanna Briggs Institute framework^28^^,^^29^ and the Preferred Reporting Items for Systematic Reviews and Meta-Analyses Extension for Scoping Reviews Checklist.^30^ The research question was based on the population, concept, and context framework^29^ and structured as follows: in adult patients receiving mechanical ventilation in the ICU (population), what is the reporting proportion of the dose prescription of early mobilization interventions (concept) in CG and IG in RCTs (context)?

### Data Sources and Searches

The searching process was performed between June 1 and 15, 2022, following the 3 stages proposed by the Joanna Briggs Institute.^29^^,^^31^ First, an initial search in PubMed was performed using some of the following keywords and MESH terms: “Physical Therapy Modalities,” “exercise,” “mechanical ventilation,” “intensive care unit,” “Critical Illness,” and “Early Ambulation.” The main concepts indicated in the titles and abstracts of relevant studies were analyzed to improve the search strategy. Second, the previously identified keywords were selected according to the relevance and availability of access by the authors and were used to refine the main search in PubMed (National Center for Biotechnology Information), Cumulative Index of Nursing and Allied Health Literature (CINAHL) Plus (EBSCO Information Services), and Web of Science (Clarivate Analytics) databases (Suppl. Tab. 1). Third, a hand search and quality control were performed to follow relevant references of the latest systematic reviews on ICU early mobilization/rehabilitation to minimize the potential loss of relevant RCTs. To include recently published RCTs, we updated the search until December 31, 2022.

### Eligibility Criteria

Following the population, concept, and context framework, we included studies comprising adult patients receiving mechanical ventilation for at least >48 hours (studies that included only patients with invasive mechanical ventilation described in methods) in the ICU (population); early mobilization modality interventions,^26^^,^^32^ including progressive mobility (passive to active mobilization, mobility activities, walking), limb NMES, leg cycling, tilt table or standing aid, and multicomponent mobilization (concept); and RCTs published from inception to June 15, 2022, including their CG (ie sham, control, and usual care groups)^33^ and IG (ie any group receiving early mobilization modality) (context). We excluded pilot or crossover studies, RCT protocols, studies in languages other than English and Spanish, pediatric studies, animal or experimental model studies, studies with poor definition of the CG, and studies without any physical functioning outcome (ie mobility, muscle strength, quality of life, and peripheral muscle mass).

### Study Selection

Two reviewers blinded from each other’s judgment (R.L.-B. and a reviewer who was not an author of this article) independently screened by title and abstract all RCTs related to early mobilization in the ICU. For studies with discrepancies, a consensus was made with a third reviewer (not an author of this article), followed by a full-text revision per the eligibility criteria. Any disagreements were resolved by that third reviewer.

### Data Extraction and Quality Assessment

A tailored data charting form was previously developed and included relevant bibliometric variables, patient characteristics, and early mobilization modality data according to the research question. The data charting form was iteratively updated as needed. In the present study, we extracted data from articles and supplementary materials, searching for all information related to early mobilization sessions (R.L.-B. and a reviewer who was not an author of this article).

Considering that all studies were physical therapy-related, we evaluated the methodological quality of the included RCTs using the Physiotherapy Evidence Database (PEDro) scale ranged from 0 to 10 points,^34^ where 0 to 3 points were considered poor, 4 or 5 points were considered fair, 6 to 8 points were considered good, and 9 or 10 points were considered excellent. Furthermore, as all trials evaluated the exercise performance (complex interventions), a total PEDro score of 8/10 was considered optimal. No studies were excluded because of methodological quality.

### Data Synthesis and Analysis

We assessed the presence of exercise prescription based on the Consensus on Exercise Reporting Template (CERT)^35^ items applicable to ICU mobilization. According to the American College of Sports Medicine recommendations on exercise doses,^36^ we extracted information according to the following applicable CERT items: item 1—detailed description of the type of exercise; item 7b—detailed description of how the exercise program progressed (progression); item 13—detailed description of dosage; and item 14b—detailed description of how exercises are tailored to the individual (individualization). Item 13 was separately assessed as daily frequency, weekly frequency, intensity, timing (starting time), and duration, as described elsewhere.^33^ The 8 CERT items were assessed as “reported” or “not reported” for the CG and IG in each RCT. We calculated the proportion of reported items/total applicable from the selected CERT items. When the information needed to define the presence or absence of a CERT item was inaccurate (eg exercise duration was between 30 and 60 minutes), we considered the mean (45 minutes). When the information of a CERT item was different in the ICU and after the ICU stay (eg session duration was 20 minutes in the ICU and 40 minutes after ICU discharge), we considered only the ICU information (20 minutes). If a CERT item was identified in one mobilization modality in studies with multicomponent mobilization, we assumed that the CERT item was reported in this RCT. For the reporting rate of the intensity or individualization of the exercise items, the items “physiotherapist criterion,” “according to tolerance and response of the patient,” or similar were not considered.

We used descriptive statistics, including counts (percentages) for categorical data and median (first–third quartiles). We summarized reporting proportion by item and across early mobilization modality classified as poor (≤50%), moderate (51–69%), or adequate (≥70%).^33^

## Results

### Data Synthesis and Study Characteristics

**Figure 1:**
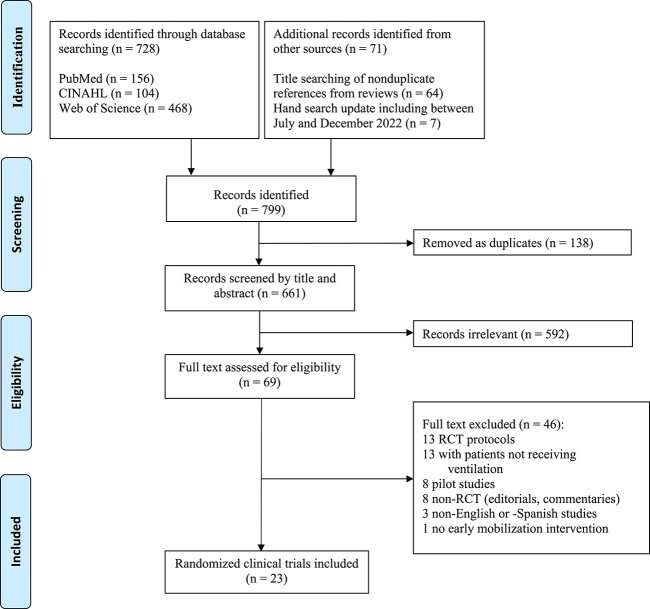
Flowchart of study selection. CINAHL = Cumulative Index of Nursing and Allied Health Literature; RCT = randomized clinical trial.

A total of 799 titles were identified. Of them, 23 RCTs comprising 2707 patients (1358 from IG and 1349 from CG) were included (Fig. 1). The majority of the RCTs were published after 2014 (73%), in European countries (48%), and in critical care/intensive care journals (57%) (Tab. 1). According to the methodological quality of the RCTs, the median PEDro scale was 6 (interquartile range [IQR] = 5–7) points, where the minimum and maximum values were 5 and 8 points, respectively (Suppl. Tab. 2). The median ICU length of stay and duration of mechanical ventilation of patients were 12 (IQR = 8–22) and 8 (IQR = 5–12) days, respectively (Tab. 2).

**Table 1:** Overview of Included Randomized Clinical Trials According to Early Mobilization Modality^*a*^

**Characteristic**	**No. (%) of** **:**					
	**Progressive Mobility Trials (*n* = 6)**	**NMES Trials (*n* = 7)**	**Leg Cycling Trials (*n* = 3)**	**Tilt Table Trials (*n* = 1)**	**Multicomponent Trials** ^*b*^ **(*n* = 6)**	**Overall Trials (*N* = 23)**
Year of publication						
2020–2022	2 (33)	2 (29)	2 (67)	0 (0)	1 (17)	7 (30)
2015–2019	3 (50)	2 (29)	1 (33)	1 (100)	3 (50)	10 (43)
2010–2014	0 (0)	3 (43)	0 (0)	0 (0)	2 (33)	5 (22)
Before 2010	1 (17)	0 (0)	0 (0)	0 (0)	0 (0)	1 (4)
Region*^c^*						
Europe	4 (67)	3 (43)	0 (0)	1 (100)	3 (50)	11 (48)
South America	1 (17)	2 (29)	2 (67)	0 (0)	1 (17)	6 (26)
Australasia	1 (17)	0 (0)	1 (33)	0 (0)	2 (33)	4 (17)
United States	3 (50)	0 (0)	0 (0)	0 (0)	1 (17)	4 (17)
Asia	0 (0)	1 (14)	0 (0)	0 (0)	0 (0)	1 (4)
Africa	0 (0)	1 (14)	0 (0)	0 (0)	0 (0)	1 (4)
Main journal scope						
Critical/intensive care	2 (33)	6 (86)	1 (33)	1 (100)	3 (50)	13 (57)
Medicine, miscellaneous	3 (50)	1 (14)	0 (0)	0 (0)	1 (17)	5 (22)
Pulmonary/respiratory	0 (0)	0 (0)	1 (33)	0 (0)	1 (17)	2 (9)
Physical therapy/rehabilitation	1 (17)	0 (0)	0 (0)	0 (0)	0 (0)	1 (4)
Anesthesiology	0 (0)	0 (0)	1 (33)	0 (0)	0 (0)	1 (4)
Physiology	0 (0)	0 (0)	0 (0)	0 (0)	1 (17)	1 (4)
Comparator group type						
Usual care/standard care	5 (83)	3 (43)	3 (100)	0 (0)	4 (67)	15 (65)
Different from usual care	1 (17)	3 (43)	0 (0)	1 (100)	2 (33)	7 (30)
Sham	0 (0)	1 (14)	0 (0)	0 (0)	0 (0)	1 (4)

^a^ Not all variables sum to 100% because of overlapping of characteristics. NMES = neuromuscular electrical stimulation.

^b^ Progressive mobility plus leg cycling (*n* = 4), progressive mobility + leg cycling + functional electrical stimulation (*n* = 1), whole-body vibration therapy + limb passive range of motion (*n* = 1).

^c^ Europe: Germany (*n* = 3), Greece (*n* = 2), France (*n* = 1), Ireland (*n* = 1), Iceland (*n* = 1), Switzerland (*n* = 1), Belgium (*n* = 1), United Kingdom (*n* = 1), Austria (*n* = 2), the Netherlands (*n* = 1). South America: Brazil (*n* = 5), Argentina (*n* = 1). Australasia: Australia (*n* = 4), New Zealand (*n* = 1). Asia: Japan (*n* = 1). Africa: Egypt (*n* = 1).

**Table 2:** Patient Characteristics of 23 Randomized Clinical Trials*^a^*

**Characteristic**	**Comparator Group**	**Intervention Group**	**Overall**
Patients enrolled, no. (%)	1349	1358	2707
Patients enrolled/study, median (IQR)	40 (19–73)	40 (22–72)	80 (40–145)
Age, y, median (IQR)*^b^*	60 (54–65)	61 (58–65)	61 (57–65)
Women, no. (%)	501 (37)	491 (36)	992 (37)
Severity of illness, median (IQR)*^b^*			
APACHE II*^c^*	21 (18–22)	20 (18–24)	20 (18–23)
SOFA*^d^*	8 (6–9)	9 (6–9)	9 (6–9)
ICU length of stay, d, median (IQR)*^b^**^,^**^c^*	13 (7–22)	12 (8–21)	12 (8–22)
Duration of mechanical ventilation, d, median (IQR)*^b^**^,^**^e^*	9 (5–12)	7 (5–10)	8 (5–12)

^a^ APACHE II = Acute Physiological and Chronic Health Evaluation II; ICU = intensive care unit; IQR = interquartile range; SOFA = Sequential Organ Failure Assessment.

^b^ Calculated with the median as reported by each study when available.

^c^ 19 trials.

^d^ 4 trials.

^e^ 17 trials.

The distribution of the early mobilization modalities of IGs of the 23 RCTs were NMES (*n* = 7), progressive mobility (*n* = 6), leg cycling (*n* = 3), tilt table (*n* = 1), and multicomponent (*n* = 6) mobilization. The majority (*n* = 13; 57%) of IGs included both the intervention and the CG therapies. The primary and secondary outcomes of the RCTs included are summarized in Supplemental Table 3.

### Reporting Proportions of CERT Exercise Prescription Items

**Figure 2:**
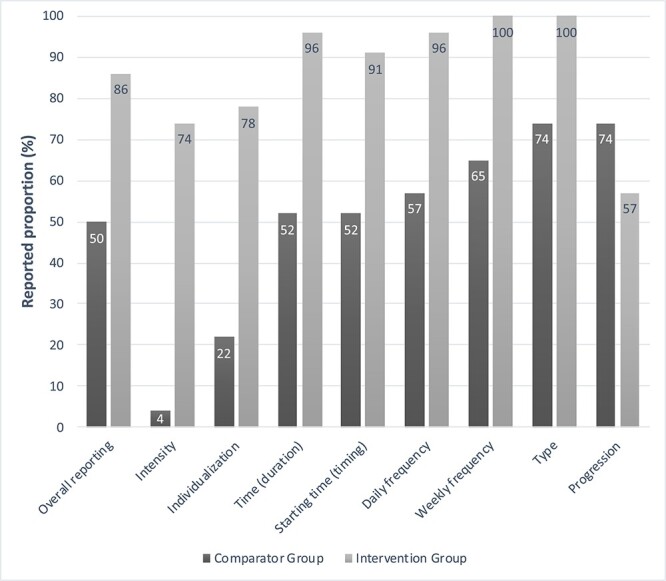
Consensus on Exercise Reporting Template (CERT) reporting of intensive care unit (ICU) early mobilization randomized clinical trials (*n* = 23).

The pooled reporting of the CERT items was “moderate” (68%) including IG and CG results. The reporting was “adequate” for IGs (86%) and almost “poor” for CGs (50%) (Fig. 2). The median proportion of reported items/total applicable was 93% (IQR = 75–99) for IGs and 54% (IQR = 29–72) for CGs. Overall, the most reported CERT items were type of exercise (100%) and weekly frequency (100%) for IGs, whereas the least reported were intensity (4%) and individualization (22%) for CG. Regardless of the group, type of exercise (CG = 74% and IG = 100%) and intensity (CG = 4% and IG = 74%) were the most and least reported items, respectively. Of the 23 RCTs, 8 (35%) IGs reported all CERT items, but no CG reported all items. For the IG, the highest and lowest reporting were identified in studies of tilt table (100%; *n* = 1) and leg cycling (79%; *n* = 3), respectively. For the CG, the highest and lowest reporting were identified in tilt table (75%; *n* = 1) and NMES (39%; *n* = 7) studies, respectively (Tab. 3).

**Table 3:** CERT Reporting in ICU Early Mobilization Randomized Clinical Trials*^a^*

**CERT Item**	**No. (%) of:**											
	**Progressive Mobility Trials (*n* = 6)**		**NMES Trials (*n* = 7)**		**Leg Cycling Trials (*n* = 3)**		**Tilt Table Trials (*n* = 1)**		**Multicomponent Trials (*n* = 6)**		**Overall Trials (*N* = 23)**	
	**CG**	**IG**	**CG**	**IG**	**CG**	**IG**	**CG**	**IG**	**CG**	**IG**	**CG**	**IG**
Starting time, timing	3 (50)	6 (100)	3 (43)	7 (100)	1 (33)	3 (100)	1 (100)	1 (100)	4 (67)	4 (67)	12 (52)	21 (91)
Daily frequency	3 (50)	5 (83)	2 (29)	7 (100)	3 (100)	3 (100)	1 (100)	1 (100)	4 (67)	6 (100)	13 (57)	22 (96)
Weekly frequency	3 (50)	6 (100)	7 (100)	7 (100)	3 (100)	3 (100)	1 (100)	1 (100)	5 (83)	6 (100)	15 (65)	23 (100)
Intensity	1 (17)	3 (50)	0	7 (100)	0	1 (33)	0	1 (100)	0	5 (83)	1 (4)	17 (74)
Time, duration	5 (83)	5 (83)	2 (29)	7 (100)	1 (33)	3 (100)	1 (100)	1 (100)	3 (50)	6 (100)	12 (52)	22 (96)
Type	4 (67)	6 (100)	3 (43)	7 (100)	3 (100)	3 (100)	1 (100)	1 (100)	6 (100)	6 (100)	17 (74)	23 (100)
Individualization	1 (17)	4 (67)	2 (29)	7 (100)	0	1 (33)	0	1 (100)	2 (33)	5 (83)	5 (22)	18 (78)
Progression	4 (67)	6 (100)	3 (43)	0	3 (100)	2 (67)	1 (100)	1 (100)	6 (100)	4 (67)	17 (74)	13 (57)
Reported items/total applicable (%)	24/48 (50)	41/48 (85)	22/56 (39)	49/56 (88)	14/24 (58)	19/24 (79)	6/8 (75)	8/8 (100)	30/48 (63)	42/48 (88)	92/184 (50)	159/184 (86)

^a^ CERT = Consensus on Exercise Reporting Template; CG = comparator group; ICU = intensive care unit; IG = intervention group; NMES = neuromuscular electrical stimulation.

### Specific Results for Reported CERT Items

When reported, the median starting time of the mobilization from ICU admission was 55 (IQR = 37–84) hours for IGs (*n* = 10) and 52 (IQR = 46–120) hours for CGs (*n* = 6), whereas other studies defined the start of the mobilization according to a relative time point (ie 48–96 hours from ICU admission, intubation, extubation, or awakening) (Tab. 4). When available, the median frequencies of mobilization were 1 (IQR = 1 or 2) time per day (*n* = 22) and 7 (IQR = 6 or 7) times per week (*n* = 23) for IGs and 1 (IQR = 1 or 2) time per day (*n* = 13) and 7 (IQR = 5–7) times per week (*n* = 15) for CGs. The intensity was mainly reported with visible/palpable muscle contraction for NMES (*n* = 9), Borg Scale and Modified Borg Scale (*n* = 6), angulation level for tilt table (*n* = 1), and maximum repetitions (*n* = 2). The median duration of the mobilization session was 30 (IQR = 20–55) minutes for IGs and 21 (IQR = 16–18) minutes for CGs. The individualization was mainly reported using functional scales (ie ICU Mobility Scale, Physical Function in ICU Test-scored, Medical Research Council Sum Score, Surgical Intensive Care Unit Optimal Mobilization Score, and Borg scale). Overall, compared with the CGs, the IGs reported starting slightly later, longer duration, more options for individualization and progression of mobilization, and similar daily and weekly frequency of mobilization. Particularly, IGs in NMES trials did not reported progression, since the parameters did not change over time.

**Table 4:** Specific Results of CERT Reporting*^a^*

**CERT Item**	**Comparator Group**	**Intervention Group**
Starting time (timing)	Median = 52 (IQR = 46–120) h from ICU admission (*n* = 6) After extubation (*n* = 1) 72 h after intubation (*n* = 1) From 96 h of mechanical ventilation (*n* = 2) On the day of ICU admission (*n* = 2) <48 h from ICU admission (*n* = 1) 96 h after ICU admission (*n* = 1) After awakening (*n* = 1)	Median = 55 (IQR = 37–84) h from ICU admission (*n* = 10) From 48 h of mechanical ventilation (*n* = 2) >48 h of mechanical ventilation (*n* = 2) At ICU admission (*n* = 4) At postoperative day 1 (*n* = 1) <48 h from ICU admission (*n* = 3) >48 hours from ICU admission (*n* = 1) 96 h after ICU admission (*n* = 1) Second day after ICU admission (*n* = 2) After awakening (*n* = 2)
Daily frequency	Median = 1 (IQR = 1 or 2) time/d (*n* = 13)	Median = 1 (IQR = 1 or 2) time/d (*n* = 22)
Weekly frequency	Median = 7 (IQR = 5–7) times/wk (*n* = 15)	Median = 7 (IQR = 6 or 7) times/wk (*n* = 23)
Intensity	Mean Borg Scale score = 13 (SD = 1.6) (*n* = 1)	Modified Borg Scale score = 3–5 (*n* = 2) Borg Scale score = 12 or 13 (*n* = 2) Borg Scale score = 11–13 (*n* = 1) Moderate to hard level of perceived exertion (*n* = 1) Visible and/or palpable muscle contraction (*n* = 9) Maximum of 6–8 repetitions (*n* = 1) 50–70% of estimated 1-repetition maximum (*n* = 1) Maximum angulation tolerated on tilt table (*n* = 1)
Time (duration)	Median = 21 (IQR = 16–18) min (*n* = 12)	Median = 30 (IQR = 20–55) min (*n* = 21) Variable, depending on protocol stage (*n* = 1)
Type	Active/passive exercises (*n* = 3) Passive/active mobilization, functional mobility (sitting on bed/chair, orthostatism, ambulation training) (*n* = 12) Passive/active mobilization, functional mobility (sitting on bed/chair, orthostatism, ambulation training) + cycling (*n* = 1)	Leg cycling (*n* = 3): passive exercise on leg cycle ergometer, 20 cycles/min (*n* = 1); leg cycling with resistance that was gradually increased during first week (*n* = 1); leg cycling that progressed from passive to active or resistance (*n* = 1) Multicomponent (*n* = 6): FES cycling and other leg cycling without FES, 35 revolutions/min (*n* = 1); muscle-activating measures, such as NMES (on 8 muscles bilaterally); and/or whole-body vibration with 1 min of stimulation, 20 Hz, and 1-min break (*n* = 1); chair transfer (passive or active/assisted) (*n* = 1); leg chair/bed cycling followed by manual passive/active limb mobilization (*n* = 3) NMES (*n* = 7): NMES on vastus lateralis, vastus medialis, and peroneus longus muscles of both lower extremities using rectangular electrodes (90 × 50 mm) (*n* = 1); NMES was only applied to the brachial biceps and vastus medialis muscles on 1 side of the body (*n* = 1); NMES using 4 rectangular electrodes (50 × 50 mm) simultaneously on quadriceps muscle of each lower extremity (*n* = 2); NMES on all parts of quadriceps muscle (rectus femoris muscle, vastus intermedius muscle, vastus lateralis muscle, vastus medialis muscle) (*n* = 1); NMES using 8 rectangular electrodes (40 × 80 mm) on biceps brachii and rectus femoris muscles (*n* = 1); NMES on quadriceps and tibialis anterior muscle surfaces bilaterally (*n* = 1) Progressive mobility (*n* = 6): intervention delivered in order to maximize early activity and muscle training, passive or active mobility/strength (resisted/free weights/body weight/elastic bands), exercises in bed or chair, active sitting or standing, transferring or marching in place (*n* = 5); walking on mobile treadmill with body weight support (*n* = 1) Tilt table (*n* = 1): passive/active mobilization, sitting in armchair and tilting (with tilt table), standing up with assistance, walking with assistance
Individualization	Categorization of mobilization levels (*n* = 1) Mobilization protocol with progression of stages (*n* = 2) ICU Mobility Scale (*n* = 1) Borg Scale (*n* = 1)	ICU Mobility Scale (*n* = 1) Borg Scale/Modified Borg Scale (*n* = 4) PFIT (*n* = 1) Visible and/or palpable muscle contraction (*n* = 8) Level of perceived exertion (*n* = 1) Percentage or number of repetition maximums (*n* = 3) Medical Research Council Sum Score, multidisciplinary daily assessment, Surgical Intensive Care Unit Optimal Mobilization Score (*n* = 1) Maximum angulation tolerated (*n* = 1)
Progression	Passive movements progressing to active according to cooperation and capacity (*n* = 4) Progression to functional activities out of bed, such as sitting, standing/active orthostatism/walking (*n* = 13) Progression criteria according to patient’s tolerance on the basis of scales and evaluations described in “individualization” and “intensity” sections	Leg cycling (*n* = 2): leg cycling in passive to active mode, resistance gradually increased Multicomponent (*n* = 4): passive to active range of movement, resistance gradually increased, bed cycle ergometer resistance increased according to capacity of patient (*n* = 2); passive/active/resisted activities + cycling that progressed to sitting, standing, and walking training (*n* = 2) NMES (*n* = 0) Progressive mobility (*n* = 6): passive/active/resisted activities that progressed to sitting, standing, and walking training (*n* = 5); walking on treadmill with increases in time and speed as tolerated (*n* = 1) Tilt table (*n* = 1): progressive verticalization protocol, progressive inclination on tilt table that progressed to standing and walking Progression criteria according to patient’s tolerance on the basis of scales and evaluations described in “individualization” and “intensity” sections

^a^ CERT = Consensus on Exercise Reporting Template; FES = functional electrical stimulation; ICU = intensive care unit; IQR = interquartile range; NMES = neuromuscular electrical stimulation; PFIT = Physical Function in Intensive Care Test.

## Discussion

Considering the existing knowledge gap on early mobilization dosing in an ICU setting, our scoping review of 23 RCTs provides relevant information on the reporting of applicable CERT items related to exercise dosing in adults receiving mechanical ventilation. We found moderate (68%) dose reporting in all studies, adequate dose reporting for IG (86%), and poor dose reporting for CG (50%). The least reported items were intensity (4%) and individualization (22%) for CG, limiting understanding of what was planned. Notably, the limited dose reporting identified in this review could, in fact, reflect the limited dose reporting in mobilized clinical practices.

We included only RCTs with all mobilization modalities (active and passive) performed on adults receiving mechanical ventilation, which adds novelty to the current literature. Recent systematic reviews on mobilization or physical rehabilitation of patients who are critically ill have included between 15 and 60 trials, depending on whether RCTs^19^ or other clinical trials were included,^18^^,^^37^ whether patients received ventilation^21^ or did not receive ventilation,^18^^,^^19^ and whether the interventions were only active^19^ or were active and passive.^18^^,^^21^ These reviews mainly focused on the effectiveness of physical rehabilitation on functional and clinical outcomes in patients who are critically ill, resulting in limited evidence for all outcomes and glimpsing relevant hypotheses such as lack of knowledge of the accurate dose mobilization in patients who are critically ill.^24^ Conversely, recent scoping and systematic reviews have also focused on the exercise prescription for mobilization interventions,^26^^,^^33^^,^^38^ identifying scarce details on mobilization description both in the intervention^26^^,^^38^ and CG.^33^ In agreement with these reviews, we found that CGs have shown worse mobilization dose reporting, with deficiencies in all CERT items and worse for exercise intensity. Remarkably, although IGs of the NMES studies had a high reporting rate, CGs of such studies had very low reporting rate for intensity and individualization. Thus, future studies should improve the dose reporting of CGs, especially when compared with patient groups receiving NMES.

We obtained a median proportion of reported CERT items of 93% for IGs and 54% for CGs comprising RCTs published until December 2022. Therefore, a recent and the largest RCT^27^ was included, thereby covering the most relevant literature. Regardless, our findings are comparable with the results of a previous scoping review with data until December 2016, where the median CERT score was 56% in 117 IGs of prospective studies.^26^ Additionally, our findings are comparable to a recent scoping review focused on the exercise reporting of CG from clinical trials, obtaining a median CERT score of 47% in studies until June 2022.^33^ This information could mean that there has been a slight improvement on the CERT reporting over time, or the inclusion of clinical trials beyond RCTs could decrease the pooled reporting.

The pursuit for the accurate mobilization dose for patients who are critically ill began many years ago.^39^^,^^40^ This has become a clear gap for researchers^24^ that still limits the reproducibility and applicability of intervention protocols in clinical practice. Accordingly, we mostly identified weaknesses in reporting the intensity, individualization, and progression of ICU mobilization, synchronous with other efforts to capture exercise dosing. Although compliance of these CERT items may be more challenging for CGs, the most common type of CG in ICU mobilization studies is usual care,^33^ suggesting that the reporting quality of these items should be a standard of care. The assessment of metabolic expenditure and amount of movement have been spread in the ICU setting using indirect calorimetry and actigraphy, respectively. Specifically, indirect calorimetry provides accurate information on oxygen consumption and carbon dioxide production during ICU activities.^41^ Its use has increased in studies of patients receiving mechanical ventilation^42^ and possibly could mitigate the lack of quantification of the intensity and consequently the individualization and progression of ICU mobilization guided by cardiopulmonary exercise testing.^41^ In addition, actigraphy is feasible to use in patients who are critically ill^2^^,^^43^^,^^44^ to capture the activity (and inactivity) of patients receiving uninterrupted mechanical ventilation throughout the ICU stay,^2^ representing another alternative to dose the mobilization of patients who are critically ill. Therefore, the emerging use of these tools in the ICU and the awareness of the underreporting of doses are identified in our scoping review, inspiring better definitions and descriptions of the ICU mobilization dose.

### Limitations

Our scoping review has limitations needing to be addressed. First, as we included RCTs in which all patients received mechanical ventilation, thus, the results should not be generalizable to other intervention studies or beyond patients receiving ventilation in the ICU, possibly, excluding relevant studies with a smaller proportion of patients receiving mechanical ventilation. Second, the CERT was not specifically designed for the ICU setting, which may not be an accurate assessment tool for scoring the mobilization of patients who are critically ill. Consequently, we selected *a priori* only items applicable to the ICU setting, minimizing the possibility of “forcing” the CERT checklist. Third, we included studies designed before 2016, where the CERT guidelines that we used to assess the completeness of the report were not yet available, yet we included the largest recently published trial of early active mobilization.^45^ Fourth, as this scoping review was initially conceived, we did not analytically compare IGs with CGs and between early mobilization modalities.

In conclusion, we identified deficits in mobilization dose reporting of ICU RCTs for adults receiving mechanical ventilation, especially for the intensity of mobilization. The dose reporting of IGs was “adequate” (86%), where one-third of IGs reported all CERT items. Additionally, the dose reporting of CGs was “poor” (22%), where no CG reported all CERT items. Considering the incomplete dose reporting of mobilization in RCTs, future studies should be designed to optimize dosage details reporting, particularly for CG.

## Supplementary Material

2023-0511_R2_Supplementary_material_R1_pe_cjt_FGS_cjt2_pzae048
