# Supplementary material for: Early Mobilization Dose Reporting in Randomized Clinical Trials With Patients Who Were Mechanically Ventilated: A Scoping Review
Source: Phys Ther. 2024 Mar 22;104(6):pzae048. doi: 10.1093/ptj/pzae048 (PMC11184528; doi:10.1093/ptj/pzae048)
Supplement: 2023-0511_R2_Supplementary_material_R1_pe_cjt_FGS_cjt2_pzae048 [file 2023-0511_r2_supplementary_material_r1_pe_cjt_fgs_cjt2_pzae048.pdf]

Supplementary Material

Early Mobilization Dose Reporting in Randomized Clinical Trials With Patients Who Were Mechanically Ventilated: A Scoping Review

Supplementary Table 1. Search Terms for the Identification of Eligible References for Scoping Review.

Database (date)	Search terms	Results
**PubMed (NCBI)** June 15, 2022	((((Early mobilization OR "Early Ambulation"[Mesh] OR Rehabilitation OR Ambulation OR "Exercise"[Mesh] OR Physical Activity OR Training OR Physical therapy OR electrical stimulation OR ergometry OR physical therapy OR rehabilitation exercise OR physiotherapy OR "Physical Therapy Modalities"[Mesh] OR exercise therapy OR bicycle OR cycle)) AND (intensive care units OR icu Intensive Care Units OR Unit Intensive Care OR "Respiration, Artificial"[Mesh] OR critical illness OR "Intensive Care Units"[Mesh])) AND (weakness OR polyneuropathy OR myopathy OR ICUAW OR myopathy OR Critical illness polyneuropathy OR muscle weakness OR paresis)) AND (Randomized Controlled Trials OR Randomized controlled trial OR "Randomized Controlled Trial" [Publication Type) **Limits:** from inception to June 15, 2022 **Language filters:** English and Spanish	156
**CINAHL (EBSCO)** June 15, 2022	(((ALL=("Early mobilization" OR "Early ambulation" OR Rehabilitation OR Ambulation OR Exercise OR Physical Activity OR Training OR Physical therapy OR electrical stimulation OR ergometry OR physical therapy OR rehabilitation exercise OR physiotherapy OR Physical Therapy Modalities OR exercise therapy OR bicycle OR cycle)) AND ALL=("intensive care units" OR "icu Intensive Care Units" OR "Unit Intensive Care" OR "artificial ventilation" OR ‘’mechanical ventilation’’	468

	OR "intensive care")) AND ALL=(weakness OR polyneuropathy OR myopathy OR ICUAW OR myopathy OR ‘’Critical illness’’ OR polyneuropathy OR ‘’muscle weakness’’)) AND ALL=("clinical trial" OR "randomized controlled trial")<br>**Limits:** from inception to June 15, 2022<br>**Type of documents filter: “**articles”<br>**Language filters:** English and Spanish	
**Web of Science**<br>June 15, 2022	("Early mobilization" OR "Early ambulation" OR Rehabilitation OR Ambulation OR Exercise OR Physical Activity OR Training OR Physical therapy OR electrical stimulation OR ergometry OR physical therapy OR rehabilitation exercise OR physiotherapy OR Physical Therapy Modalities OR exercise therapy OR bicycle OR cycle) AND ("intensive care units" OR "icu Intensive Care Units" OR "Unit Intensive Care" OR "artificial ventilation" OR ‘’mechanical ventilation’’ OR "intensive care") AND (weakness OR polyneuropathy OR myopathy OR ICUAW OR myopathy OR ‘’Critical illness’’ OR polyneuropathy OR muscle weakness OR paresis) AND (MH "Clinical Trials+") OR (MH "Randomized Controlled Trials+")<br>**Limits:** from inception to June 15, 2022<br>**Language filters:** English and Spanish	104

Supplementary Table 2. Methodological Quality of Randomized Clinical Trials Using the PEDro Scale.

Early Mobilization modality	Author, year of publication	PEDro score
Progressive mobility trials	Schweickert et al, 2009[1]	8
	Moss et al, 2016[2]	6
	Schaller et al, 2016[3]	5
	Amundadottir et al, 2019[4]	4
	Kwakman, 2022[5]	6
	TEAM study, 2022[6]	8
NMES trials	Routsi et al, 2010[7]	4
	Rodríguez et al, 2012[8]	6
	Abu-Khaber et al, 2013[9]	5
	Fischer et al, 2016[10]	6
	Koutsioumpa et al, 2018[11]	4
	Nakanishi et al, 2020[12]	6
	Campos, 2022[13]	5
Leg cycling trials	Machado et al, 2017[14]	5
	Nickels et al, 2020[15]	7
	Araujo de Azevedo et al, 2021[16]	5
Tilt-table trials	Sarfati et al, 2018[17]	7
Multicomponent trials	Dantas et al, 2012[18]	4
	Denehy et al, 2013[19]	7
	Eggmann et al, 2018[20]	7
	Hickmann et al, 2018[21]	6
	Wollersheim et al, 2019[22]	4
	Berney et al, 2021[23]	6
Overall, Median [IQR]		6 [5–7]

NMES = neuromuscular electrical stimulation, IQR = interquartile range, PEDro = Physiotherapy Evidence Database

Supplementary Table 3. Brief Description of Randomized Clinical Trials Outcomes on Early Mobilization of Mechanically Ventilated Patients.

Author, year of publication	Early Mobilization Modality	Primary Outcome	Secondary Outcomes	Outcomes in favor of the mobilization	Outcomes not in favor of mobilization
**Hodgson, 2022**[6]	**Progressive mobility**	Number of days that patients were alive and out of the hospital at day 180.	Death at day 180; number of ventilator-free days; days out of the ICU from randomization to day 28; Functional outcomes in survivors at day 180; score on EQ-5D-5L utility score; score on EQ Visual Analog Scale; median score on Barthel Index of ADL; median score on IADL; median score on WHODAS 2.0.		Number of days that patients were alive and out of the hospital at day 180; patients with ≥1 adverse event potentially due to mobilization; Death at day 180; ventilator-free days, and ICU-free days at day 28; EQ-5D-5L utility score; score on EQ Visual Analog Scale; Median score on Barthel Index of ADL; IADL; WHODAS 2.0.
**Campos, 2022**[13]	**NMES**	Functional status at ICU using FSS-ICU.	FSS-ICU first day awake and at ICU discharge and hospital discharge; MRC-SS in ICU and hospital discharge; ICUAW occurrence by MRC-SS; PFIT first day awake, and at ICU and hospital discharge; Barthel index at ICU and hospital discharge; EQ-5D-3L at ICU and hospital discharge; frequency of delirium using CAM-ICU.	FSS-ICU at ICU and hospital discharge; ICU-AW occurrence; hospital LOS.	Barthel index; ICU LOS; days on mechanical ventilation; quality of life; and frequency of delirium.

**Kwakman, 2022**[5]	**Progressive mobility**	Number of days to independent ambulation by FAC.	Maximum walking distance by 2MWT; extremities muscle strength at 7 days after inclusion at hospital discharge and when reaching the study endpoint by MRC-SS; perceived exertion after physiotherapy interventions (6–20 Borg Scale); De Morton Mobility Index; Hospital length of stay; symptoms of post-traumatic stress; patient satisfaction at hospital discharge.	Hospital length of stay.	Time to independent ambulation; Max walking distance; De Morton Mobility Index; MRC-SS; symptoms of post-traumatic stress; patient satisfaction at hospital discharge.
**Berney, 2021**[23]	**Multicomponent**	Quadriceps muscle strength at hospital discharge (isometric torque, measured in Newton meters); Prevalence of cognitive impairment at 6 months (neuropsychological battery of tests)	All-cause mortality, occurrence and duration of delirium, manual muscle testing, hand grip strength, physical function in ICU Test score; Functional Status Score for the ICU; SPPB; 6 MWT; Katz Index of independence ADL; Lawton's IADL; Hospital Anxiety and Depression Scale; Impact of Events Scale-Revised; SF-36 v2 and 5-level D6EQ-5D.		Cognitive, physical, and psychological outcomes.
**Araujo de Azevedo, 2021**[16]	**Leg cycling**	PCS score 3 and 6 months after randomization obtained from the medical outcomes (SF-36)	ICUAW occurrence by handgrip strength; duration of mechanical ventilation; the ICU length of stay; ICU and hospital mortality.	PCS Score; ICUAW occurrence; ICU mortality; hospital mortality.	ICU and hospital length of stay; duration of mechanical ventilation.
**Nickels, 2020**[15]	**Leg cycling**	Muscle atrophy at Day 10 post-study enrolment in rectus femoris by cross-sectional area.	Rectus femoris and vastus intermedius thickness were also measured by sonographers at baseline, day 3, day 7, day 10 post-study enrolment, and 7 days following ICU discharge; MRC-SS; handgrip strength; FSS-ICU; all measured at ICU discharge and 1 week following ICU discharge; a single 6MWT measured;<br>1 week following ICU discharge;		Muscle atrophy; MRC-SS and ICU-AW occurrence at day 5; IMS at discharge from the ICU; ventilator and ICU-free days (28 days after ICU admission); length of hospitalization.

			IMS at ICU discharge.		
**Nakanishi, 2020**[12]	**NMES**	Muscle thickness and cross-sectional area of the biceps brachii and rectus femoris muscles from days 1 to 5.	MRC-SS and ICUAW occurrence at day 5; IMS at discharge from the ICU; ventilator and ICU-free days (28 days after ICU admission); length of hospitalization; and percentage change in amino acid level.	Limb muscle thickness; cross-sectional area/BCAA levels on day 3; Glycine levels on days 3 and 5 and proline levels on day 3 were lower in the NMES than in the control group.	MRC score; ICU-AW occurrence; IMS at discharge from the ICU.
**Wollersheim, 2019**[22]	**Multicomponent**	MRC-SS and handgrip dynamometry on awakening, at ICU discharge, and at a 12 month in-hospital follow-up; FIM at ICU discharge and at a 12-month follow-up; 6MWT at a 12 month in-hospital follow-up; Molecular analyses by biopsy of the vastus lateralis muscles.	Gene expression and protein content; myosin content; pathways of protein synthesis; protein degradation; and local inflammation.	Prevents muscle atrophy.	Muscle strength: Functional mobility assessed by the locomotive component of the FIM score at ICU discharge; 6MWT; gene expressions of key mediators of the protein-degradation pathway; gene expressions of key mediators of the protein-degradation pathway; inflammatory cytokines IL-6, expression for TNF.
**Amundadottir, 2019**[4]	**Progressive mobility**	Duration of mechanical ventilation; ICU and hospital lengths of stay.	HRQoL 4 weeks before ICU admission at ICU discharge, at hospital discharge, and at 3, 6 and 12 months after ICU discharge; SF-36v2; 6MWT; MRC-SS; ICU acquired weakness using MRC-SS; Modified Barthel Index.		Duration of mechanical ventilation; ICU and hospital lengths of stay; physical function measurements.

**Hickmann, 2018**[21]	**Multicomponent**	Regulation of protein degradation/synthesis pathways during the first week.	Preservation of the muscle fiber cross-sectional area; presence of exercise-induced muscle inflammation; restoration of neuromuscular function by measuring electrophysiology values and muscle strength; safety and tolerance of the intervention.	Catabolic ubiquitin-proteasome pathway, muscle atrophy F-box and muscle ring finger-1 messenger RNA, were reduced at day 7; muscle fiber cross-sectional area was preserved by exercise; molecular regulations suggest that the excessive activation of autophagy due to septic shock was lower in the intervention group; markers of anabolism and inflammation were not modified by the intervention, which was well tolerated by the patients.	
**Sarfati, 2018**[17]	**Tilt-table**	MRC-SS at ICU discharge	-Muscular recovery during the ICU stay (change in MRC score from baseline to ICU discharge) and MRC score at hospital discharge; ICU and hospital stay lengths, use of sedation, NMB, or corticosteroids, and mechanical ventilation days.	Hospital mortality	The MRC sum scores at ICU and hospital discharge; ICUAW occurrence; ICU and hospital stay lengths, use of sedation, NMB, or corticosteroids, and mechanical ventilation days.
**Koutsioumpa, 2018**[11]	**NMES**	Incidence of histologically diagnosed myopathy on the 14th ICU Day.	MRC-SS on the 14th ICU Day; Duration of mechanical ventilation; length of ICU stay; ICU, 28-day; 1-year mortality.		Histologic evidence of myopathy; ventilator-free days; ICU mortality; ICU stay; MRC-SS on day 14.
**Eggmann, 2018**[20]	**Multicomponent**	6MWT, FIM at hospital discharge.	FIM and muscle strength (MRC-SS) at ICU discharge, handgrip strength; quadriceps muscle strength measured with a handheld dynamometer; limitations in range of motion were recorded for shoulder flexion, elbow flexion and extension, fist closure, hip flexion, knee flexion and extension, and foot dorsiflexion.<br>-functional mobility the Timed “Up & Go” was performed at hospital discharge; time on mechanical ventilation, ICU and hospital length of stay along with achieved ICU mobility; SF-36 6 months after hospital discharge.		For primary outcomes were not significantly different between the two groups.<br>-ICUAW at ICU discharge; SF-36; limitations in range of motion; FIM; MRC-SS; MRC-SS at ICU discharge, handgrip strength; quadriceps muscle strength measured with a handheld dynamometer were not significantly different between the two groups.

**Machado, 2017**[14]	**Leg cycling**	MRC-SS before and after the implementation of the study protocol.	Duration of mechanical ventilation; length of ICU stays; length of hospital stays.	MRC-SS	Duration of mechanical ventilation; ICU length of stay; hospital length of stay.
**Moss, 2016**[2]	**Progressive mobility**	CS-PFP-10: 1, 3 and 6 months after study enrolment	ICU- and hospital-free days at day 28; discharge to home; all-cause mortality at day 28; and institution-free days at day 90 and day 180; The Five Times Sit to Stand Test, TUG, The Berg Balance Test, and SF-36v2.		CS-PFP-10: 1, 3, and 6 months after study enrolment; Five Times Sit to Stand Test, TUG, the Berg Balance Test, and SF-36v2.
**Fischer, 2016**[10]	**NMES**	Muscle layer thickness of the quadriceps muscle of both thighs using two-dimensional B- mode ultrasound, measured on postoperative day 1, every other day until ICU discharge and at hospital discharge; muscle strength using MRC-SS; ICUAW using MRC-SS; bilateral grip strength	FIM; TUG; SF-12 were evaluated at hospital discharge, the average mobility level proposed by Brown.	Patients in the NMES group had a 4.5 times higher slope in recovering muscle strength during the ICU stay than patients in the control group.	Muscle layer thickness of the quadriceps muscle; grip strength; changes in all functional outcomes from preoperative day to ICU or hospital discharge.

**Schaller, 2016**[3]	**Progressive mobility**	SOMS level during ICU stay; length of stay on ICU; mmFIM; functional capacity for locomotion and transfers (eg, moving oneself from a bed to a chair) each on a scale of 1 (near complete dependence) to 4 (complete independence).	MRC-SS once a week; ICUAW using MRC-SS; SF-36 at 3 months after hospital discharge.	Length of stay on ICU; SOMS level during ICU stay; MRC-SS; mmFIM; The mobility-related functional independence scores at hospital discharge; mobility-related outcomes.	ICUAW occurrence; SF-36 at 3 months after hospital discharge.
**Denehy, 2013**[19]	**Multicomponent**	6MWT; ICUAW; HRQoL; SF-36v2; TUG; PFIT.			ICUAW; HRQoL; SF-36v2; PFIT; 6MWT.
**Abu-Khaber, 2013**[9]	**NMES**	-MRC-SS; duration of weaning from mechanical ventilator.		Duration of mechanical ventilation; MRC-SS at day 4 and all through till day 21.	MRC-SS at day 2 and day 3; The MRCS at day 28.
**Rodriguez, 2012**[8]	**NMES**	Arm and leg circumferences were measured at the middle line every 48 hours; biceps thickness; MRC-SS.		Arm circumference; MRC-SS.	Biceps thicknesses.

**Dantas, 2012**[18]	**Multicomponent**	Respiratory muscle strength was indirectly assessed every 3 days (PImax and PEmax) at ICU discharge; peripheral muscle strength using MRC-SS.	Duration of mechanical ventilation.	MRC-SS.	Duration of mechanical ventilation; PEmax and Pimax.
**Routsi, 2010**[7]	**NMES**	CIPNM occurrence using MRC-SS for awakening until ICU discharge; duration of weaning from mechanical ventilation and intensive care unit stay were recorded.	Weaning period.	CIPNM occurrence; weaning period; number of days off the ventilator.	
**Schweickert, 2009**[1]	**Progressive mobility**	Number of patients returning to independent functional status at hospital discharge.	Duration of delirium; ventilator-free day; greatest walking distance at hospital discharge; MRC-SS at hospital discharge; handgrip strength at hospital discharge; independent ADL total at ICU discharge.	Return to independent functional status at hospital discharge; Duration of delirium; ventilator-free days; walking distance at hospital discharge.	Independent ADLs total at ICU discharge; MRC-SS at hospital discharge; handgrip strength at hospital discharge.

Abbreviations: EQ-5D-5L = EuroQol Group 5-Dimension Self-Report Questionnaire; WHODAS 2.0 = World Health Organization Disability Assessment Schedule; ADL = activities of daily living; IADL = instrumental activities of daily living; IQR = Interquartile range; FSS-ICU= Functional Status Score for the Intensive Care Unit; MRC-SS = Medical Research Council Sum Score; CAM-ICU = Confusion Assessment Method for the Intensive; LOS = length of stay; EM = early mobilization; 2MWT = 2 minutes walking test; FAC = Functional Ambulation Categories; SF-36 = 36-item short-form health survey; PCS = physical component summary; ICUAW = ICU-acquired weakness; IMS = ICU Mobility Scale; 6MWT = 6 minutes walking test; IL6 = interleukin 6; TNF = Tumor necrosis factor; NMB = Neuromuscular blocking; MV = mechanical ventilation; CS-PFP-10 = Continuous-Scale Physical Functional Performance Test; SF-36v2 = 36-item short-form health survey 2 version; SOMS = Surgical ICU Optimal Mobilization Score; FIM = Mini-modified functional independence measure score; HRQoL= Health-related quality of life; TUG = Timed Up and Go; PFIT = The Physical Function in ICU Test; APACHE II = Acute Physiology and Chronic Health disease Classification System II; PImax = maximal inspiratory pressure; PEmax = maximal expiratory pressure; CIPNM: critical illness polyneuromyopathy

References

1. Schweickert WD, Pohlman MC, Pohlman AS, et al. Early physical and occupational therapy in mechanically ventilated, critically ill patients: a randomised controlled trial. *The Lancet*. 2009;373(9678):1874-1882. doi:10.1016/S0140-6736(09)60658-9

2. Moss M, Nordon-Craft A, Malone D, et al. A Randomized Trial of an Intensive Physical Therapy Program for Patients with Acute Respiratory Failure. *Am J Respir Crit Care Med*. 2016;193(10):1101-1110. doi:10.1164/rccm.201505-1039OC

3. Schaller SJ, Anstey M, Blobner M, et al. Early, goal-directed mobilisation in the surgical intensive care unit: a randomised controlled trial. *The Lancet*. 2016;388(10052):1377-1388. doi:10.1016/S0140-6736(16)31637-3

4. Amundadottir OR, Jónasdóttir RJ, Sigvaldason K, et al. Effects of intensive upright mobilisation on outcomes of mechanically ventilated patients in the intensive care unit: a randomised controlled trial with 12-months follow-up. *Eur J Physiother*. 2021;23(2):68-78. doi:10.1080/21679169.2019.1645880

5. Kwakman RCH, Voorn EL, Horn J, et al. Steps to recovery: Body weight-supported treadmill training for critically ill patients: A randomized controlled trial. *J Crit Care*. 2022;69:154000. doi:10.1016/j.jcrc.2022.154000

6. TEAM Study Investigators and the ANZICS Clinical Trials Group. Early Active Mobilization during Mechanical Ventilation in the ICU. *New England Journal of Medicine*. 2022;387(19):1747-1758. doi:10.1056/NEJMoa2209083

7. Routsi C, Gerovasili V, Vasileiadis I, et al. Electrical muscle stimulation prevents critical illness polyneuromyopathy: A randomized parallel intervention trial. *Crit Care*. 2010;14(2). doi:10.1186/cc8987

8. Rodriguez PO, Setten M, Maskin LP, et al. Muscle weakness in septic patients requiring mechanical ventilation: Protective effect of transcutaneous neuromuscular electrical stimulation. *J Crit Care*. 2012;27(3):319.e1-319.e8. doi:10.1016/j.jcrc.2011.04.010

9. Abu-Khaber HA, Abouelela AMZ, Abdelkarim EM. Effect of electrical muscle stimulation on prevention of ICU acquired muscle weakness and facilitating weaning from mechanical ventilation. *Alexandria Journal of Medicine*. 2013;49(4):309-315. doi:10.1016/j.ajme.2013.03.011

10. Fischer A, Spiegl M, Altmann K, et al. Muscle mass, strength and functional outcomes in critically ill patients after cardiothoracic surgery: Does neuromuscular electrical stimulation help? The Catastim 2 randomized controlled trial. *Crit Care*. 2016;20(1). doi:10.1186/s13054-016-1199-3

11. Koutsioumpa E, Makris D, Theochari A, et al. Effect of Transcutaneous Electrical Neuromuscular Stimulation on Myopathy in Intensive Care Patients. *American Journal of Critical Care*. 2018;27(6):495-503. doi:10.4037/ajcc2018311

12. Nakanishi N, Oto J, Tsutsumi R, et al. Effect of Electrical Muscle Stimulation on Upper and Lower Limb Muscles in Critically Ill Patients: A Two-Center Randomized Controlled Trial. *Crit Care Med*. 2020;48(11):E997-E1003. doi:10.1097/CCM.0000000000004522

13. Campos DR, Bueno TBC, Anjos JSGG, et al. Early Neuromuscular Electrical Stimulation in Addition to Early Mobilization Improves Functional Status and Decreases Hospitalization Days of Critically Ill Patients. *Crit Care Med*. 2022;50(7):1116-1126. doi:10.1097/CCM.0000000000005557

14. Machado A dos S, Pires-Neto RC, Carvalho MTX, Soares JC, Cardoso DM, Albuquerque IM de. Effects that passive cycling exercise have on muscle strength, duration of mechanical ventilation, and length of hospital stay in critically ill patients: a randomized clinical trial. *Jornal Brasileiro de Pneumologia*. 2017;43(2):134-139. doi:10.1590/s1806-37562016000000170

15. Nickels MR, Aitken LM, Barnett AG, et al. Effect of in-bed cycling on acute muscle wasting in critically ill adults: A randomised clinical trial. *J Crit Care*. 2020;59:86-93. doi:10.1016/j.jcrc.2020.05.008

16. de Azevedo JRA, Lima HCM, Frota PHDB, et al. High-protein intake and early exercise in adult intensive care patients: a prospective, randomized controlled trial to evaluate the impact on functional outcomes. *BMC Anesthesiol*. 2021;21(1). doi:10.1186/s12871-021-01492-6

17. Sarfati C, Moore A, Pilorge C, et al. Efficacy of early passive tilting in minimizing ICU-acquired weakness: A randomized controlled trial. *J Crit Care*. 2018;46:37-43. doi:10.1016/j.jcrc.2018.03.031

18. Dantas CM, Silva PFDS, Siqueira FHT de, et al. Influence of early mobilization on respiratory and peripheral muscle strength in critically ill patients. *Rev Bras Ter Intensiva*. 2012;24(2):173-178. http://www.epistemonikos.org/documents/883a351a89865b70d6611a09590a8b92b8918802 NS -

19. Denehy L, Skinner EH, Edbrooke L, et al. Exercise rehabilitation for patients with critical illness: a randomized controlled trial with 12 months of follow-up. *Crit Care*. 2013;17(4):R156. doi:10.1186/cc12835

20. Eggmann S, Verra ML, Luder G, Takala J, Jakob SM. Effects of early, combined endurance and resistance training in mechanically ventilated, critically ill patients: A randomised controlled trial. *PLoS One*. 2018;13(11). doi:10.1371/journal.pone.0207428

21. Hickmann CE, Castanares-Zapatero D, Deldicque L, et al. Impact of very early physical therapy during septic shock on skeletal muscle: A randomized controlled trial. *Crit Care Med*. 2018;46(9):1436-1443. doi:10.1097/CCM.0000000000003263

22. Wollersheim T, Grunow JJ, Carbon NM, et al. Muscle wasting and function after muscle activation and early protocol-based physiotherapy: an explorative trial. *J Cachexia Sarcopenia Muscle*. 2019;10(4):734-747. doi:10.1002/jcsm.12428

23. Berney S, Hopkins RO, Rose JW, et al. Functional electrical stimulation in-bed cycle ergometry in mechanically ventilated patients: A multicentre randomised controlled trial. *Thorax*. 2021;76(7):656-663. doi:10.1136/thoraxjnl-2020-215093
